# Development and Longitudinal Analysis of Plan-Based Streamlined Quality Assurance on Multiple Positioning Guidance Systems With Single Phantom Setup

**DOI:** 10.3389/fonc.2021.683733

**Published:** 2021-06-16

**Authors:** Shun Zhou, Junyu Li, Yi Du, Songmao Yu, Meijiao Wang, Hao Wu, Haizhen Yue

**Affiliations:** ^1^ Key Laboratory of Carcinogenesis and Translational Research (Ministry of Education/Beijing), Department of Radiation Oncology, Peking University Cancer Hospital & Institute, Beijing, China; ^2^ Institute of Medical Technology, Peking University Health Science Center, Beijing, China

**Keywords:** quality assurance, positioning guidance, robotic couch, surface imaging, image guided radiotherapy

## Abstract

**Purpose:**

This study was to propose and validate an efficient and streamlined quality assurance (QA) method with a single phantom setup to check performances of patient positioning guidance systems including six-degree-of-freedom (6DoF) couch, X-ray modalities (kV–kV, MV–MV and CBCT), optical surface imaging system (AlignRT), lasers and optical distance indicator (ODI).

**Methods and Materials:**

The QA method was based on a pseudo-patient treatment plan using the AlignRT cube phantom. The cube was first randomly set up on the couch, and the initial position offsets were acquired by AlignRT and CBCT. The cube was restored to its reference position by 6DoF couch shift, during which the couch motion accuracy and tracking performances of AlignRT and CBCT were derived. After that, the residual offsets were acquired by kV–kV, MV–MV and AlignRT to derive the isocenter discrepancies. Finally, the laser alignment and ODI values were visually inspected. The QA procedure had been internally approved as a standard weekly QA test, and the results over 50 weeks were longitudinally analyzed for clinical validation.

**Results:**

The 6DoF couch motion errors as well as the tracking errors of AlignRT were sub-millimeter and sub-degree, and no deviation over 1 mm or 1 deg was identified. The ROI mode of isocenter (ISO) in AlignRT exhibited more consistent results than the centroid (CEN). While the isocenter discrepancy between CBCT and kV–kV was negligible, the maximal discrepancies between CBCT and MV–MV were 0.4 mm in LNG and 0.3 deg in PITCH. The isocenter discrepancies between CBCT and AlignRT were <0.5 mm in translation and <0.3 deg in rotation. For AlignRT, the isocenter discrepancies between the DICOM and SGRT references were about 0.6 mm in VRT, 0.5 mm in LNG and 0.2 deg in PITCH. As the therapists became familiar with the workflow, the average time to complete the whole procedure was around 23 min.

**Conclusions:**

The streamlined QA exhibits desirable practicality as an efficient multipurpose performance check on positioning guidance systems. The stability, tracking performance and isocenter congruence of the positioning guidance systems have been fully validated for all clinical image guidance RT application, even SRS/SBRT, which requires the strictest tolerance.

## Introduction

Tremendous developments of linear accelerator and guidance systems in modern precision radiotherapy (RT) enable the delivery of high radiation doses to tumors with improved adjacent organs-at-risk (OARs) sparing (1). Since these dose distributions are often characterized by steep dose gradients, to safeguard patients as well as maximize the treatment benefits, it is pivotal to ensure the accuracy as well as inter-fraction consistency in patient positioning (2).

As conventional routine, therapists set up patients by aligning skin markers to laser crosses, and then tumor localization is verified *via* in-room X-ray imaging modalities such as kV/MV images and cone-beam CT (CBCT). Acquired images are registered to planning CT to determine the position and tumor displacement, which are then corrected by robotic couch shift.

To ensure patient positioning accuracy, comprehensive quality assurance (QA) is imperative on all the systems involved in the procedure (3). According to AAPM TG-104 (4), TG-142 (5) and TG-147 (6), performances should be periodically checked on room lasers, crosshair, robotic couch, X-ray imaging systems and etc. However, some QA programs are tedious and time-consuming to complete with repeated phantom displacements and manual measurements. To enhance the QA efficiency for machine performance, streamlined QA is clinically essential and various methods have been proposed (7–10). For instance, Varian has released a commercial module named Machine Performance Check (MPC) (Varian, CA, USA) for its high-end LINAC systems. Once a specific fiducial-embedded cylindrical phantom is set up, MPC streamlines multiple mechanic and beam consistency checks by acquiring series of MV or kV images (11–14). Since the procedure is highly efficient, MPC is typically scheduled on each day before treatment.

Over the past decade, new technologies that use stereo computer vision to image patient surface in three-dimension have been developed. Thanks to the intrinsic advantages of high frame-rate, large field-of-view and radiation-free over in-room X-ray imaging, patients can be well aligned as in simulation with reduced errors and improved consistency (15). The study of Stanley et al. (16) showed that compared with the conventional setup approach, optical surface imaging based setup method significantly improved patient positioning accuracy by mitigating the initial errors detected by CBCT in multiple sites including breast, chest, abdomen and etc.

Moreover, patients can be dynamically monitored with beam-hold control on radiation delivery, which effectively safeguards patients in case of accidental movements. As the clinical benefits of frameless SRS become widely recognized (17–19), growing institutions have installed surface guidance systems towards surface guided radiotherapy (SGRT) (20).

Despite the fact that performance checks on SGRT and linac systems are off-the-shelf, the congruence between SGRT systems and existing positioning guidance systems (laser, CBCT, etc.) are not included in the current performance check programs. In the meantime, these programs are commercially licensed by vendors, which are neither independent nor cross-platform accessible. Therefore, it is sensible to propose a unified QA program that integrates all patient positioning guidance systems, including SGRT system, lasers, planar kV/MV, CBCT and robotic couch.

Inspired by the success of MPC, this study aims to propose a streamlined QA program which can be efficiently performed with a single phantom setup to check performances of multiple patient positioning guidance systems and their congruence. The key initiative we had in mind when designing this QA procedure is to be practical, which means the procedure should be streamlined, multi-purpose, highly efficient, easy to perform, independent, cross-platform, and provide native data archiving. It is important to note, while we used the same phantom, similar treatment platform and QA concept with the work by Kang et al. (9), the key QA focus as well as the workflow were different in nature.

In the rest of this work, the overall procedure, data acquisition and analysis are first detailed. Then, the clinical application at our institution over 50 weeks is presented and discussed. Finally, the QA program and results are discussed with key conclusions summarized.

## Materials and Methods

### Treatment and Patient Positioning Guidance Systems

This work was performed on a VitalBeam Treatment Delivery System (Varian, CA, USA), which is equipped with a six degree-of-freedom (6DoF) robotic couch, kV OBI, MV EPID and on-board CBCT. Driven by TrueBeam 2.7, the VitalBeam is able to perform 6DoF registration on CBCT (3D-to-3D) as well as kV–kV and MV–MV orthogonal pairs (2D-to-3D). Along with the VitalBeam, an AlignRT (version 5.1.1, VisionRT, London, UK) optical surface guidance system is installed and interlinked to the VitalBeam for beam control. Besides quick daily system tests such as MPC, periodical QA activities are performed on these positioning guidance systems to guarantee the performance. For instance, monthly QA and MV calibration of AlignRT are scheduled on the first week of each month for senior therapists to perform. For every four weeks, kV imager and EPID are recalibrated in service mode, and CBCT image quality reproducibility is evaluated by a vendor-delivered CatPhan^®^ 504 phantom.

At our institution, the integrated system is used clinically to treat breast, H&N and brain-metastasis patients. For metastasis treatment, patients are typically immobilized with double-sided open-faced masks, and aligned to reference positions under AlignRT guidance. During dose delivery, patients are dynamically monitored in real-time to hold the beam once patient movements are out-of-threshold. Note that the recommended SRS/SBRT tolerance in AAPM TG-142 is followed at our institution as the threshold for metastasis patient movement (1 mm in translation and 1 deg in rotation), which is referred to as the clinical tolerance on system performance.

### Phantom and QA Plan Preparation

**Figure 1:**
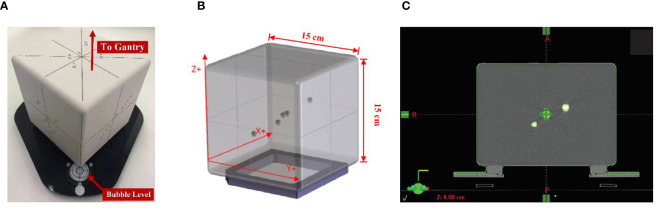
AlignRT cube phantom used in this study: **(A)** the cube phantom was placed on a levelling plate with adjustable screws, and set up with one edge towards the LINAC gantry to facilitate AlignRT surface imaging; **(B)** five radiopaque spheres with a diameter of 7.5 mm were asymmetrically embedded into the cube with one at the isocenter; **(C)** the plan isocenter was carefully placed on the central marker of the cube.

The AlignRT cube phantom was used in this study. As shown in **Figure 1**, the phantom is an opaque polystyrene cube with side length as 15 cm and visible to AlignRT for optical surface imaging. Inside the cube, five alumina ceramic spheres are embedded with one at the center and the other four off-center placed. The high-density spheres exhibit high contrast to kV and MV X-ray. On each side of the cube, thin black lines indicating the cube center are clearly marked in vertical and horizontal directions.

The proposed QA program was treatment plan based. In plan preparation, the cube phantom was first scanned on our Sensation Open CT-Sim (Siemens Healthineers AG, Germany) using the default head protocol (kVp = 120, slice thickness = 1.5 mm). The CT images were then transferred to our Eclipse (Varian, CA, USA) treatment planning system (TPS) to create a pseudo-patient (named as SGRT at our institution) as well as a treatment plan. In the treatment plan, the five radio-opaque spheres were contoured as markers for registration, and what’s more the isocenter should be carefully placed to the phantom center, i.e., the center of the central sphere as shown in **Figure 1C**. In the meantime, image verifications of CBCT, kV–kV and MV–MV pairs were also added in the plan.

### Overall Procedure and Data Acquisition

**Figure 2:**
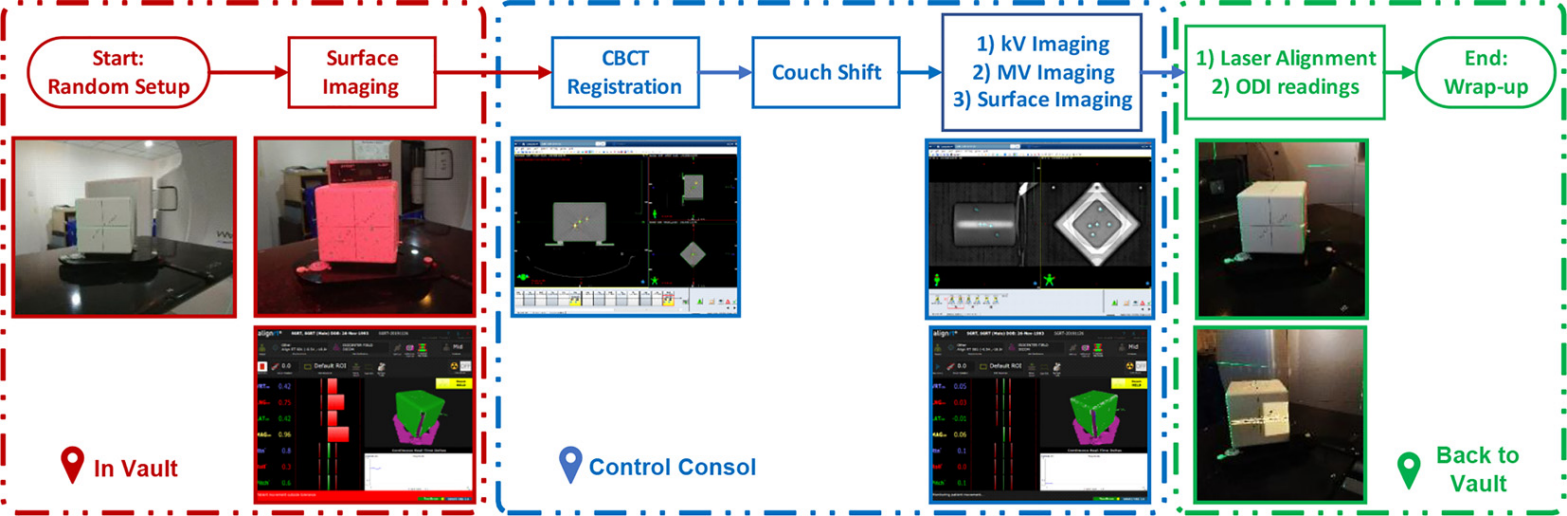
Step-by-step procedure of the streamlined QA program: from cube set-up to wrap-up.

The standard QA procedure is illustrated in **Figure 2**, which is very similar to the clinical routine for patient treatment. Generally, the procedure is streamlined in seven steps as:

***Step 1***: After the pseudo-patient plan is opened on the linac console, a therapist enters the vault and sets up the cube phantom. The cube is randomly set up on the couch, but should be within 5 cm of the isocenter.
***Step 2***: the therapist starts surface monitoring on AlignRT to acquire the initial offset from the reference position.
***Step 3***: the therapist exits the vault and performs a CBCT scan on the linac console.
***Step 4***: the CBCT to planning CT registration is performed, and the robotic couch is shifted accordingly the registration result. It is important to note that at this moment the cube has been corrected to the reference position as in planning CT, and as a result the residual 6DoF errors given by guidance systems should be as small as possible.
***Step 5***: kV–kV and MV–MV pairs are sequentially performed and registered to reference images. In the same process, AlignRT is started to monitor the cube in real time. As mentioned above, all these errors given by kV–kV, MV–MV and AlignRT should be within respective tolerance levels.
***Step 6***: the therapist re-enters the vault to read the optical distance indicator (ODI) values at gantry 0 and gantry 90. Also, the lasers are evaluated by inspecting its congruence to the phantom mark lines, and phantom leveling in pitch and roll are checked by a leveling gadget.
***Step 7***: the therapist wraps up the cube to complete the QA procedure.

It is worth noting that the related images and positioning errors in surface and X-ray guidance as well as couch shifts are all archived in AlignRT and TPS respectively, which can be off-line reviewed and analyzed retrospectively. Only the ODI values are manually documented.

### Reference Surface and ROI Mode Selection

The software version of AlignRT used in this study is 5.1.1, which allows alternatives in reference surface and ROI mode. Besides the default reference surface (denoted as DICOM) derived from the CT-Sim outline structure, an optical reference surface (denoted as SGRT) can be acquired by captures from the stereo cameras. During preparation, the cube was first aligned to the planning CT isocenter *via* CBCT registration, and then carefully adjusted to achieve ideal leveling in ROLL and PITCH. Thereafter, the SGRT surface was acquired and used in the following. Note that for the same ROI, AlignRT offers two modes in calculating the positioning errors: isocenter (ISO) and centroid (CEN). As the naming indicates, the reference point for positioning error calculation is designated to the AlignRT isocenter in ISO and to the specific ROI mass center in CEN. As a consequence, there were four combinations available to choose for positioning guidance: DICOM-ISO, DICOM-CEN, SGRT-ISO and SGRT-CEN. To investigate the effect of reference surface and ROI type selection on positioning errors, the positioning errors in each combination were recorded by reference and ROI type switching.

### Longitudinal Analysis of System Performances

Since the cube plan was first internally approved as a standard weekly QA test, the proposed QA program has been regularly performed by therapists at our institution for more than 1 year. Each time the test was performed, the overall time between therapist setting up and wrapping up the cube was recorded. For ease of illustration, we analyzed the results over the first 50 weeks to evaluate the errors in AlignRT, 6DoF robotic couch, kV–kV, MV–MV, lasers and ODI.

As the first part of the proposed QA program, the CBCT-guided couch shift was applied to restore the cube to its reference position as in CT-Sim. The error of the 6DoF couch was defined as the discrepancy from the actual applied shift to the CBCT-guided shift:

$$\Delta_{couch}=\left(CouchPosition_{pre}-CouchPosition_{post}\right)-CBCT$$

Similarly, the tracking error of AlignRT is defined as

$$\Delta_{RefROI}=(RefROI_{pre}-RefROI_{post})-CBCT$$

where *RefROI* denotes the selection in reference surface and ROI mode, i.e., DCM-ISO, DCM-CEN, SGRT-ISO and SGRT-CEN.

After the CBCT-guided couch shift, the congruence of kV–kV, MV–MV and AlignRT were evaluated by the residual errors. In addition, the alignment of room lasers to the cube marker lines were visually inspected, and the ODI at gantry 0 and 90 were manually measured.

Note that the errors of each system with respect to the CBCT isocenter were statistically analyzed with nonparametric Wilcoxon signed ranked tests, where p <0.01 indicates rejection to null hypothesis at the 99% confidence level.

## Results

### Couch Motion Accuracy and Tracking Performance of Imaging Modalities

**Figure 3:**
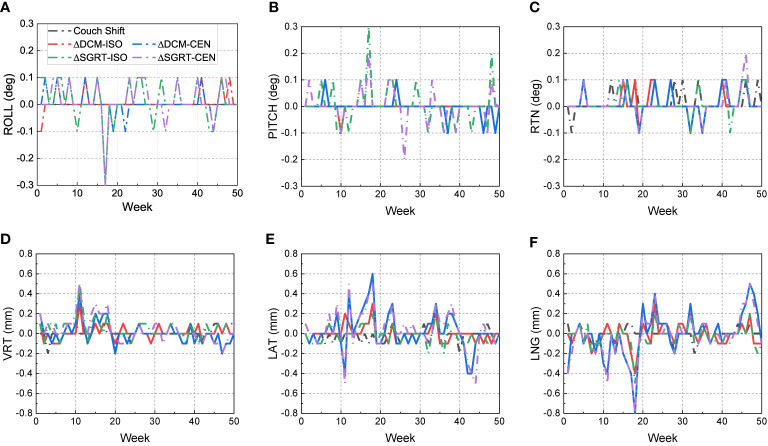
Discrepancies from CBCT-guided shifts to the actual couch shifts and AlignRT trackings over 50 weeks. The trends are listed in six dimensions respectively: **(A)** ROLL (deg), **(B)** PITCH (deg), **(C)** RTN (deg), **(D)** VRT (mm), **(E)** LAT (mm) and **(F)** LNG (mm).

**Figure 4:**
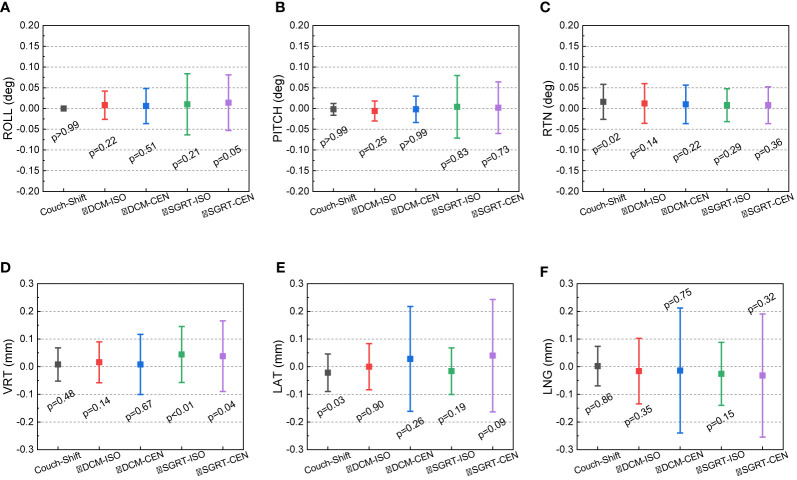
Mean, standard deviation (error bars) of the discrepancies from the CBCT-guided shifts to the actual couch shifts and AlignRT trackings. The mean and stand deviation values are listed in 6 dimensions respectively: **(A)** ROLL (deg), **(B)** PITCH (deg), **(C)** RTN (deg), **(D)** VRT (mm), **(E)** LAT (mm) and **(F)** LNG (mm).

**Figure 3** shows the 50-week discrepancies of the actual couch shifts and AlignRT tracked shifts in respect to the CBCT-guided shifts in six directions: A) ROLL (deg), B) PITCH (deg), C) RTN (deg), D) VRT (mm), E) LAT (mm) and F) LNG (mm). The corresponding mean, standard deviation (error bars) and the nonparametric Wilcoxon p-value in each direction are shown in **Figure 4**.

For each rotational direction of ROLL, PITCH and RTN, the discrepancies were generally zero with the maximum as 0.3 deg. The mean values are very close to zero with p-values >0.05. The largest standard deviation was in SGRT-ISO along PITCH as 0.075 deg. Considering the random noise-related uncertainty of AlignRT is 0.1 deg (20), the error level is as small as negligible (p = 0.83).

For each translational direction of VRT, LAT and LNG, the discrepancies fluctuated around zero with the maximum as 0.8 mm. The p-values are all more than 0.05, except the SGRT-ISO results in VRT (p < 0.01). Since the random noise-related uncertainty of AlignRT in translational is 0.2 mm (6, 20), the error (0.04 ± 0.101 mm) is not clinically significant. Also, it is clearly shown that the maximum 0.8-mm discrepancies exhibited only when the ROI mode of CEN were used, i.e., DCM-CEN and SGRT-CEN. Also, the error bars of DCM-CEN and SGRT-CEN were much larger than the ISO-mode counterparts. After we reviewed the data records in TPS and AlignRT, it is found that the CEN-related large discrepancies are highly correlated with large couch shifts (>8 mm in translation or >0.7 deg in rotation). Since the cube was first randomly set up, this indicates that the couch shift-based measurements in the ISO mode is more credible than those in the CEN mode for the proposed QA procedure, which will be validated in future work. Besides, the negligible discrepancies and compact error bars of the couch shifts indicates the 6DoF robotic couch is well maintained and operates in excellent performances.

For PITCH, VRT and LNG, relatively larger systematic isocenter discrepancies can be clearly identified. For MV–MV, the isocenter discrepancies in LNG and PITCH was around 0.42 mm and 0.2 deg. Considering the poor contrast in MV images, we believe this could be attributed to the MV-MV registration algorithm. For kV–kV, while the fluctuations between −0.3 and 0.3 deg can be seen in PITCH, the overall discrepancies in PITCH, VRT and LNG are within 0.1 deg and 0.2 mm. For AlignRT with DICOM as reference, isocenter discrepancy was almost zero in PITCH, 0.4 mm in VRT and 0.3 mm in LNG. For AlignRT with SGRT reference, isocenter discrepancy was about 0.2 deg in PITCH, 0.2 mm in VRT and 0.2 mm in LNG.

### Isocenter Discrepancies

**Figure 5:**
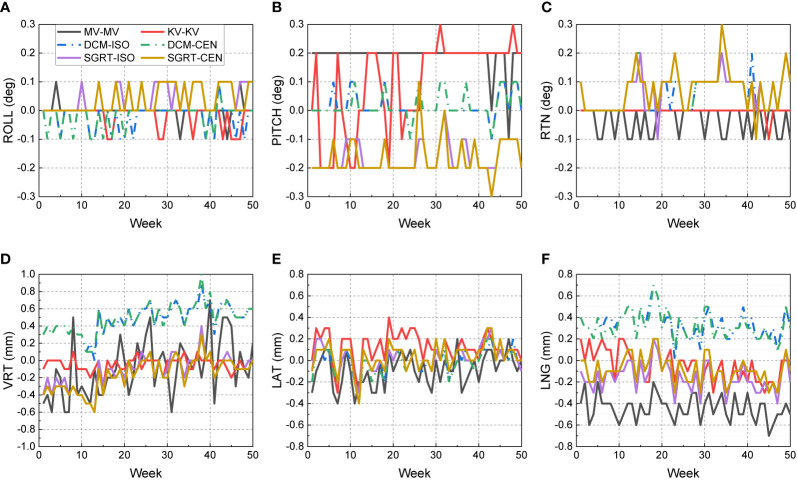
Discrepancies from the CBCT isocenter to kV–kV, MV–MV and AlignRT isocenters over 50 weeks. The trends are listed in 6 directions respectively: **(A)** ROLL (deg), **(B)** PITCH (deg), **(C)** RTN (deg), **(D)** VRT (mm), **(E)** LAT (mm) and **(F)** LNG (mm).

**Figure 6:**
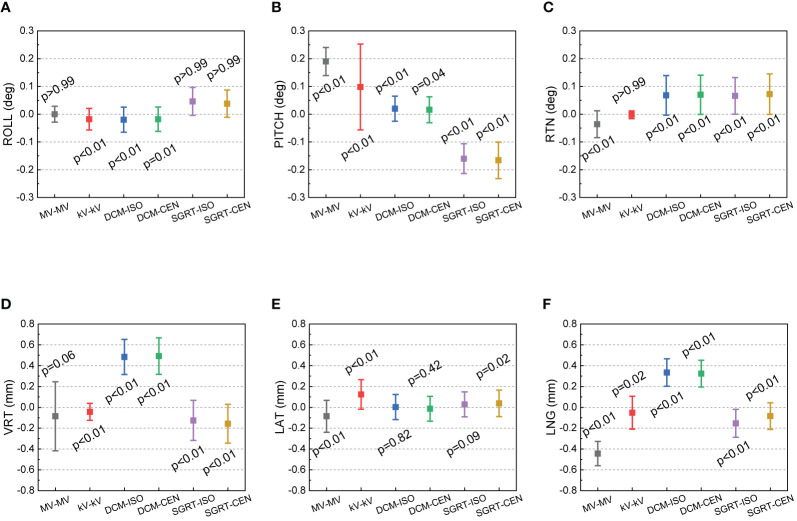
Mean, standard deviation (error bars) of the discrepancies from the CBCT isocenter to kV–kV, MV–MV and AlignRT isocenters over 50 weeks. The mean and stand deviation values are listed in six directions respectively: **(A)** ROLL (deg), **(B)** PITCH (deg), **(C)** RTN (deg), **(D)** VRT (mm), **(E)** LAT (mm) and **(F)** LNG (mm).

**Figure 5** shows the 50-week discrepancies from the CBCT isocenter for MV–MV, kV–kV and AlignRT in six directions: **(A)** ROLL (deg), **(B)** PITCH (deg), **(C)** RTN (deg), **(D)** VRT (mm), **(E)** LAT (mm) and **(F)** LNG (mm). The corresponding mean, standard deviation (error bars) and nonparametric Wilcoxon p-value in each direction are shown in **Figure 6**.

For rotational directions of ROLL and RTN, the maximum discrepancy from the kV–kV and MV–MV isocenters to the CBCT isocenter was 0.1 deg, and the maximum discrepancy between AlignRT and CBCT was 0.3 deg. While slight isocenter discrepancies between CBCT and other imaging modalities can be identified, the deviations are less than 0.1 deg. A similar trend is also shown in LAT, where the approximately 0.1-mm isocenter discrepancies between CBCT and other imaging modalities can be identified. As a treatment plan-based QA program, the discrepancies might be induced from any upstream processes, such as cube setup errors during CT-Sim and manual isocenter designation errors in TPS.

Also, for AlignRT, the isocenter discrepancy between the DICOM reference surface and the SGRT surface was about 0.6 mm in VRT, 0.5 mm in LNG and 0.2 deg in PITCH. We believe these could be majorly attributed to two parts. On the one hand, while the DICOM reference surface was derived in TPS *via* a predefined threshold (default as −350 HU in Eclipse), the SGRT surface was acquired from camera captures. Compared with SGRT, the DICOM surface inwardly shrunk a little bit (the extent of which is unclear yet and will be explored in future). On the other hand, while the inferior ridge of the cube structure was somehow lost (as shown in **Figure 2**) during DICOM files importing, the capture SGRT surface lost vision of the superior ridge of the cube probably for light reflection. As a consequence, the coupled effect between the shrinking surface in DICOM and the asymmetry ROI in VRT and LNG likely led to the identified isocenter shifts between the DICOM and SGRT reference surfaces. In the meantime, since PITCH, VRT and LNG are highly correlated in rigid registration, the isocenter deviation in PITCH could be derivative from those in VRT and LNG. In addition, other processes during plan preparation might also induce some errors, such as noise and partial volume effect (slice thickness = 1.5 mm) in CT imaging, CT-Sim couch sag, auto-registration errors from CBCT to planning CT, and etc.

### Laser Alignment and ODI

For the lasers, although the alignment to mark lines was visually inspected, it is interesting to note that we did find a gradual drift in one of the lasers. Without this regular QA test, it would be much hard to identify this problem.

As for ODI, while the expected SSD values in TPS were 92.6 cm at gantry 0 and 89.7 cm at gantry 90, the observed ODI readings were 92.5 ± 0.1 cm at gantry 0 and 89.6 ± 0.1 cm at gantry 90.

### Time Efficiency

**Figure 7:**
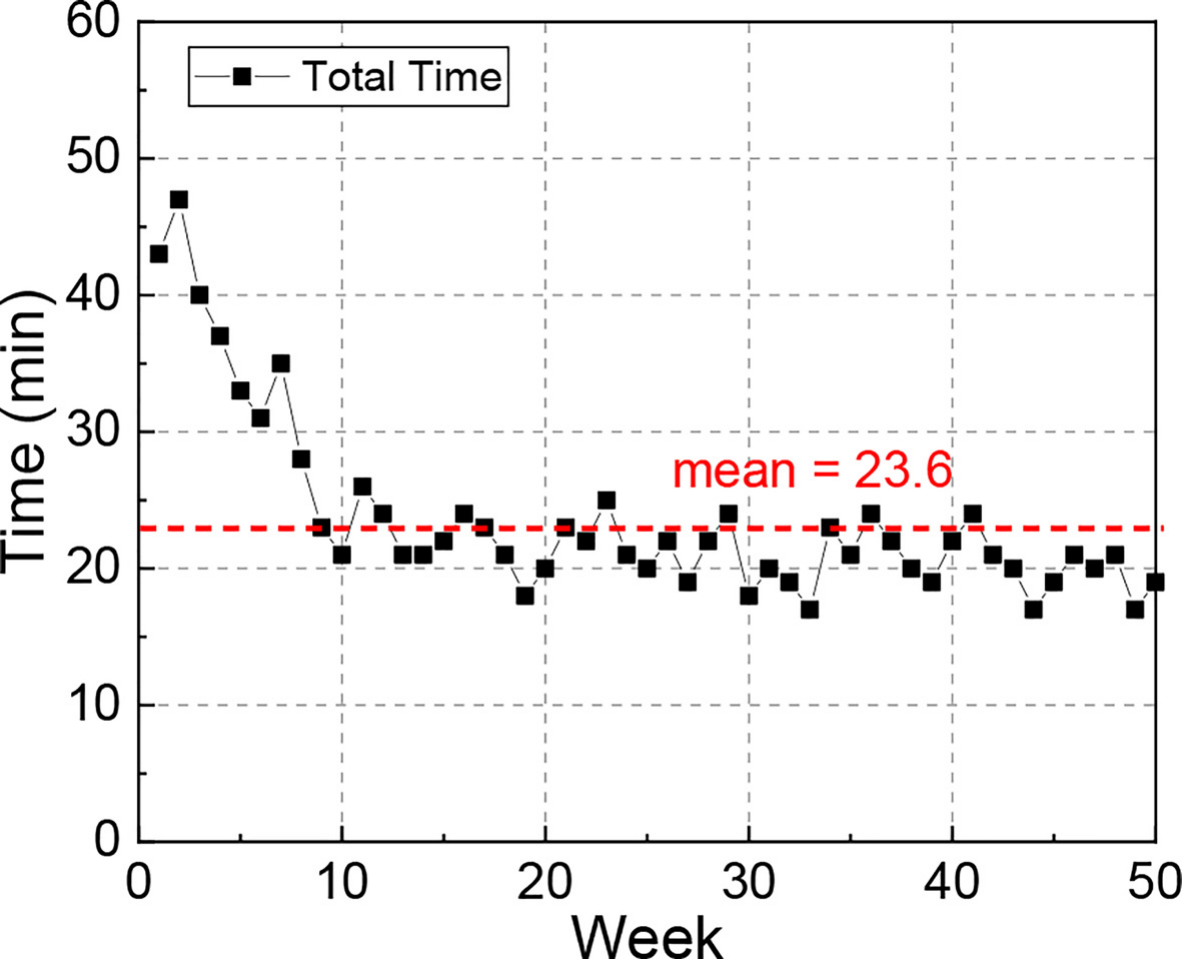
Plot of total time required to complete the QA test over 50 weeks. This end-to-end time was recorded from therapists starting to set up the phantom and finishing wrapping up the phantom.

The plot of required time vs. week is illustrated in **Figure 7**. When the QA test was first approved into clinic, it took around 40 min for the therapists to complete the whole procedure. As the therapists became familiar with the workflow, the time gradually reduced to about 20 min, and the average time over the 50 tests was 23.6 min.

## Discussion

In this study an efficient QA program was proposed to check the tracking performance of multiple patient positioning systems and the isocenter congruence of the X-ray and surface guidance system (AlignRT). Despite the fact that this study was performed on a VitalBeam LINAC and AlignRT surface guidance system, the program was treatment plan based and in-house prepared in an end-to-end approach. Therefore, derivative programs for other linacs and surface guidance systems can be easily developed using third-party phantoms that contain registration fiducials.

Besides cross-platform, other important features of the proposed QA program can be summarized as: 1) versatile for performance and congruence check on multiple systems, including robotic couch, surface guidance system, X-ray imaging modalities (kV–kV, MV–MV and CBCT), lasers and ODI; 2) streamlined with single and random phantom setup, which makes the procedure efficient and therapist-friendly to perform; 3) auto-archiving of test results, which facilitates off-line review and trend analysis.

From the 50-week QA results at our institution, the couch shift accuracy and the shift tracking accuracy of AlignRT were sub-millimeter and sub-degree, and no drift over 1 mm or 1 deg were identified. As for the ROI mode in AlignRT, the results in the ISO mode were more consistent than the CEN mode when large couch shifts were applied, indicating that the ISO mode is recommended in further application.

The isocenter discrepancies between CBCT and other imaging modalities were investigated. While the MV–MV isocenter exhibits over 0.4 mm in LNG and 0.3 deg in PITCH, the kV–kV isocenter discrepancy is as small as negligible. As for AlignRT, the isocenter discrepancies between the DICOM and SGRT references are about 0.6 mm in VRT, 0.5 mm in LNG and 0.2 deg in PITCH.

Despite of these, the isocenter discrepancies from CBCT to other X-ray modalities and AlignRT are all sub-millimeter and sub-degree, which is consistent with the AAPM TG-147 (6) and within the recommended SRS/SBRT tolerance in AAPM TG-142 (5).

Considering the QA program was performed in an end-to-end approach, to some extent the discrepancy indicates the level of end-to-end errors at our institution. Since the magnitude is as small as comparable to that of the linac gantry mechanical isocenter, this indicates the excellent accuracy of AlignRT in surface tracking and localization. Nonetheless, it is still an interesting issue to minimize the end-to-end discrepancy, which we will explore in further work.

Compared with the Varian MPC test, this program aims to check the performance on multiple positioning guidance systems. On the one hand, these guidance systems are critical to safeguard patients in everyday setup and tumor localization, especially prevailing surface-guided open-faced mask frameless SRS and markerless patient setup. On the other hand, the systems are indispensable for some QA activities. For example, lasers and ODI are typically used as reference to align tools.

As the major strength of our work, especially when compared with Kang’s excellent work (9) for isocenter congruence QA of multiple imaging systems *via* W-L test in TrueBeam Developer Mode, the proposed QA program in this study is fully in Clinical Mode without additional function or feature requirement on treatment machines, which can be easily implemented with minimal cost by average institutions on similar platforms.

As a key limitation of the proposed QA program, the couch rotation performance for non-coplanar treatment was not included. The major reason is that at our institution non-coplanar delivery technologies such as Dynamic Conformal Arc and HyperArc have not yet been commissioned into clinical use yet. Since the QA program is treatment plan based, couch rotation testing for non-coplanar treatments can be easily incorporated when necessary.

## Conclusions

A streamlined QA program on positioning systems has been developed with a single phantom setup. As an effective complement to comprehensive QA activities, this program is very practical in taking multiple-purpose performance check on positioning guidance systems including robotic couch, surface imaging, X-ray modalities, lasers and ODI. Since its approval, the proposed program has been weekly performed weekly for more than one year. The stability, tracking performance and isocenter congruence of these positioning guidance systems have been fully validated for all clinical image guidance RT application, even SRS/SBRT, which requires the strictest tolerance.

## Data Availability Statement

The original contributions presented in the study are included in the article/supplementary material. Further inquiries can be directed to the corresponding authors.

## Author Contributions

SZ designed the QA workflow and wrote the manuscript. JL and SY performed the experimental and optimized the workflow. YD statistically analyzed the results. MW designed the QA plan and analyzed the results. HY and HW revised the manuscripts. All authors contributed to the article and approved the submitted version.

## Funding

This work was supported in part by the Beijing Natural Science Foundation (Nos. 1212011, 1202009), National Natural Science Foundation of China (No. 12005007), National Key Research and Development Project (No. 2019YFF01014405), Capital’s Funds for Health Improvement and Research (No. 2018-4-1027), Ministry of Education Science and Technology Development Center (No. 2018A01019), Beijing Municipal Administration of Hospitals Incubating Program (No. PX2019042), Science Foundation of Peking University Cancer Hospital (No. 2021-14, 2021-1).

## Conflict of Interest

The authors declare that the research was conducted in the absence of any commercial or financial relationships that could be construed as a potential conflict of interest.
